# 5-ALA-assistant automated detection of lymph node metastasis in gastric cancer patients

**DOI:** 10.1007/s10120-020-01044-w

**Published:** 2020-02-11

**Authors:** Tatsuya Matsumoto, Yasutoshi Murayama, Hisataka Matsuo, Kengo Okochi, Naotaka Koshiishi, Yoshinori Harada, Hideo Tanaka, Tetsuro Takamatsu, Eigo Otsuji

**Affiliations:** 1 Division of Digestive Surgery, Department of Surgery, Kyoto Prefectural University of Medicine, 465 Kajiicho, Kawaramachi-Hirokoji, Kamigyo-ku, Kyoto, 6028566 Japan; 2 Department of Pathology and Cell Regulation, Kyoto Prefectural University of Medicine, 465 Kajiicho, Kawaramachi-Hirokoji, Kamigyo-ku, Kyoto, 6028566 Japan; 3 Ushio Inc., 6409 Moto-Ishikawa-cho, Aoba-ku, Yokohama, Kanagawa 2250004 Japan; 4 Department of Medical Photonics, Kyoto Prefectural University of Medicine, 465 Kajiicho, Kawaramachi-Hirokoji, Kamigyo-ku, Kyoto, 6028566 Japan

**Keywords:** 5-Aminolevulinic acid, Protoporphyrin IX, Lymph node metastasis, Gastric cancer

## Abstract

**Background:**

5-aminolevulinic acid (5-ALA) has been utilized for cancer diagnosis as a fluorescence probe. We have reported the feasibility of 5-ALA-induced protoporphyrin IX (PpIX) fluorescence for detecting lymph node (LN) metastasis in gastrointestinal malignancies. However, a major barrier to the fluorescence diagnosis has been that the evaluation has been highly dependent on the observers. In this study, we examined the validity of a developed device for automated detection without subjectivity.

**Methods:**

Gastric cancer patients who received oral administration of 5-ALA (20 mg/kg) prior to surgery were enrolled. For a total of 323 LNs obtained from 64 patients, the diagnostic results of the device were compared to those of conventional histopathological examination based on hematoxylin-and-eosin-stained slides. The accuracy with the device was compared to that of stereoscopic detection with conventional fluorescence microscopy for 211 LNs from 42 patients. We used two types of image processing that we previously developed to eliminate autofluorescence of background tissues: differential and ratio methods.

**Results:**

For detection of metastasis in 323 LNs, the areas under the receiver operating characteristic curves with the differential method and ratio method were 0.921 and 0.909, respectively. The sensitivity, specificity, and accuracy with the differential method were 78.0%, 96.8%, and 94.4%; while those with the ratio method were 78.0%, 96.1%, and 93.8%, respectively. In 211 LN analysis, the diagnostic accuracy with the device was comparable to that of stereoscopic examination.

**Conclusion:**

Our device for automated detection of LN metastasis using 5-ALA can be a useful tool for intraoperative diagnosis.

**Electronic supplementary material:**

The online version of this article (10.1007/s10120-020-01044-w) contains supplementary material, which is available to authorized users.

## Introduction

Gastric cancer is the fifth most common malignant tumor and the third leading cause of cancer-related mortality worldwide [1]. Along with the marked improvement of therapeutic techniques, there is a growing emphasis on not only the curability but also the postoperative quality of life. To achieve this goal, sentinel node navigation surgery (SNNS) has been employed in the treatment of patients with gastric cancer. Recently, sentinel node (SN) basin dissection, which is a selective lymphadenectomy for en bloc dissection of lymphatic tissue including lymph nodes (LNs) and vessels, has been reported for the early gastric cancer surgery [2]. In this strategy, if SNs are negative for metastasis, function-preserving gastrectomy with SN basin dissection, including local resection, segmental gastrectomy, pylorus-preserving gastrectomy, and proximal gastrectomy would be possible for individual patients.

To attain these benefits, accurate intraoperative LN metastasis diagnosis is necessary. Intraoperative diagnosis of LN metastasis is generally performed based on frozen section analysis, but the sensitivity is limited since only a few slides are examined per specimen [3]. In addition, freezing artifacts deteriorate the quality of the sample [4], which makes it difficult for pathologists to diagnose in some cases. In recent years, diagnostic methods for LN metastasis of gastric cancer using molecular biological techniques such as RT-PCR [5] and one-step nucleic acid amplification [6] have been reported. These methods are superior in that they are more sensitive than conventional pathological diagnostic methods, and are capable of evaluating the entire LN at once, not just a few slides. However, the major drawback is that no diagnostic specimen remains due to the process of sample lysate. The possibility of false-positive results caused by contaminated epithelial tissue should be considered in these methods [7]; thus, it is difficult to confirm the presence of contamination. In addition, it takes more than 30 min to obtain diagnostic results. Thus, there is a need for a reliable rapid intraoperative method to assess the presence of metastases of freshly excised LNs.

Therefore, we have focused on ex vivo fluorescence diagnosis using 5-aminolevulinic acid (5-ALA), and previously reported its feasibility for detecting LN metastases in gastrointestinal cancers [8–10]. When 5-ALA is administered in the human body exogenously, it is rapidly metabolized to heme in normal cells; while protoporphyrin IX (PpIX), a heme precursor, selectively accumulates in cancer cells due to the altered activity of key enzymes in the heme synthetic pathway (e.g., increased activity of porphobilinogen deaminase, and decreased activity of ferrochelatase) [11, 12]. PpIX emits a strong red fluorescence peaking at around 635 nm on blue light excitation [13]; thus, cancer diagnosis is enabled by detecting the fluorescence.

However, a major barrier in the fluorescence diagnosis has been that the evaluation has been highly dependent on the observers. We consider that the variability is due to several factors. First, even if PpIX fluorescence is derived from cancer cells, a sample that exhibits relatively weak fluorescence intensity may be recognized as non-metastatic. Second, PpIX accumulates nonspecifically in inflammatory sites [14, 15] and emits red fluorescence, making it difficult to accurately distinguish fluorescence derived from cancer tissue in conventional macroscopic detection. In addition, in human tissues, there are abundant endogenous fluorophores such as collagen and flavins that emit strong autofluorescence in the broad fluorescence spectrum peaking at around *λ* = 520 nm under blue light excitation [16–18]. The spectrum also contains red fluorescence components, which can lead to misdiagnosis.

Therefore, we developed a device that automatically detects LN metastasis by quantitatively evaluating the PpIX fluorescence intensity, while eliminating tissue autofluorescence. We applied two types of methods to eliminate tissue autofluorescence, which we have previously reported: differential method [19] and ratio method [20]. We explored the potential usefulness of the newly developed device for automated detection of LN metastasis.

## Materials and methods

### Clinical study

A clinical trial was performed at University Hospital of Kyoto Prefectural University of Medicine, between October 2015 and August 2016. This clinical trial is registered on UMIN Clinical Trial Registry (registry number, UMIN 000018504). All clinical experiments were conducted with the approval of the Ethics Committees of Kyoto Prefectural University of Medicine (approval No. ERB-C**-**364**)** as well as in accordance with guidelines from the committees, and conform to the provisions of the Declaration of Helsinki. Patients who were diagnosed with gastric cancer preoperatively were consecutively included in this study after having signed the informed consent form. Exclusion criteria were as follows: history of porphyria, history of allergy, renal or hepatic insufficiency, gastrointestinal obstruction, intake of hypericin-containing medication, histamine H2 receptor antagonist or proton pump inhibitor. We prospectively enrolled 66 patients, two of whom underwent operation at Matsushita Memorial Hospital. Two patients were excluded because of passage obstruction due to pyloric stenosis and failure to take 5-ALA, respectively. Finally, a total of 323 LNs obtained from 64 patients were automatically analyzed with the developed device. Of them, 211 LNs from 42 patients were also observed with conventional fluorescence microscopy. The details of the clinicopathological characteristics of the 64 patients are shown in Table 1.

**Table 1:** Clinicopathological characteristics of the enrolled patients

Clinicopathological features of enrolled patients	*n* = 64
Age (range)	67.6 (35–87)
Sex	
Male	45 (70.3%)
Female	19 (29.7%)
Average number of examined lymph nodes (range)	5.0 (1–8)
Operative procedure	
Distal gastrectomy	42 (65.6%)
Proximal gastrectomy	8 (12.5%)
Total gastrectomy	13 (20.3%)
Exploratory laparotomy	1 (1.6%)
Histological type	
Differentiated	34 (53.1%)
Undifferentiated	30 (46.9%)
Tumor depth (*T*)	
*T*1	37 (57.8%)
*T*2	13 (20.3%)
*T*3	12 (18.8%)
*T*4	2 (3.1%)
Pathological stage (UICC)	
Stage I	43 (67.2%)
Stage II	13 (20.3%)
Stage III	6 (9.4%)
Stage IV	2 (3.1%)

### 5-ALA administration

5-ALA hydrochloride (ALAGLIO) was received from SBI Pharma Co., Ltd. (Tokyo, Japan). 5-ALA hydrochloride, dissolved in 20 ml of 50% glucose solution at a concentration of 20 mg/kg of body weight, was orally applied to each patient 3 h before induction of general anesthesia. Patients were protected from direct sunlight for 24 h after 5-ALA administration to avoid photosensitization.

### Sample preparation

We targeted regional LNs which were dissected during surgery. Since this study was designed to preliminarily validate the measurement system of this device, we collected and analyzed dissected LN samples, regardless of the degree of cancer staging and without targeting sentinel LNs in early gastric cancer. The LNs suspected of metastasis were preferentially selected. When there was no LNs suspected of metastasis, the ones which have relatively large size were selected. The LNs were immediately brought into the laboratory while avoiding exposure. The LNs were sliced with the thickness of 2 mm at the center using a fine razor, while ones less than 2 mm in diameter were transected in half. After fluorescence image acquisition of the cut surfaces, the observed samples were fixed with 10% formalin. Hematoxylin-and-eosin (H&E)-stained slides of serial sections to the fluorescence observation planes were prepared; then experienced pathologists, who did not know information about the results of fluorescence analysis, made histopathological diagnosis.

### Optical design

#### Conventional fluorescence microscopy

A stereoscopic fluorescence microscope (SZX-16; Olympus) was used to acquire fluorescence images. A mercury lamp (U-HGLGPS; Olympus) was employed as light source for fluorescence excitation. The wavelength of excitation light was set at 405 nm using an optical filter (D405/20x; Chroma). Fluorescence from the sample was collimated with an objective lens (SDFPLAPO × 0.5; Olympus), transmitted through a long-pass filter (HQ430LP; Chroma) at wavelength longer than 430 nm, and imaged on a color charge-coupled device (CCD) camera (DP73; Olympus) with the exposure time of 1 s.

#### Device for automated detection of lymph node metastasis

**Fig. 1:**
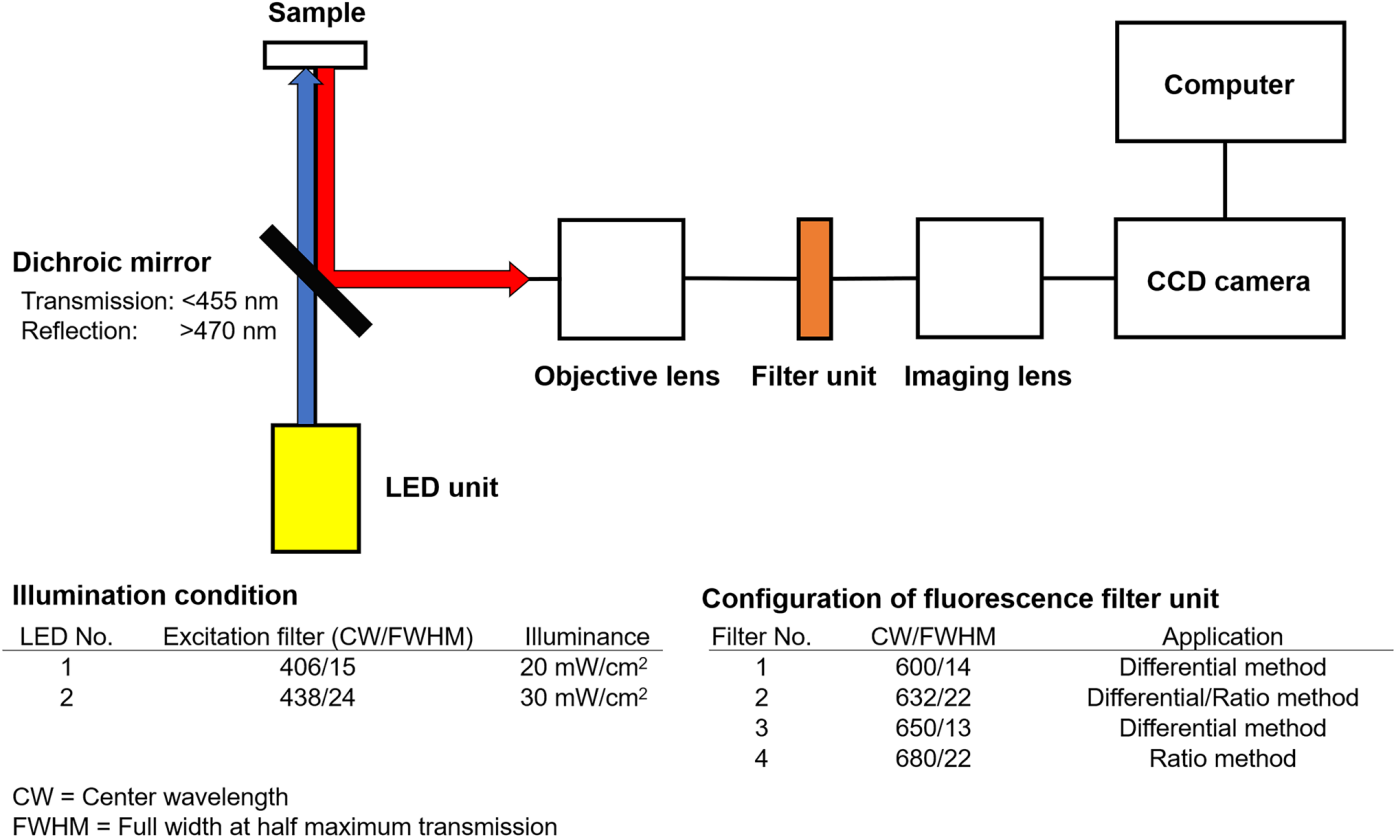
Schematic view of the device for automated detection of LN metastasis

We built a device equipped with an optics enabling automated acquisition of florescence images of a sample. A schematic view of the optical design is shown in Fig. 1. We placed the sample in a cassette, and the sample was illuminated by the light emitting from an LED unit. The unit consists of two tunable LEDs (CBT-90-UV and CBT-90-B, Luminus), which have excitation filters of either 405 nm or 435 nm (FF01-406/15, FF02-438/24, Semrock), respectively. Fluorescence emitting from the sample was reflected by the dichroic mirror at wavelengths longer than 470 nm, collimated with an objective lens (MVPLAPO × 0.63, Olympus), and imaged on a monochrome CCD camera (Lt665R, Lumenera). An optical filter unit was placed at the position between the objective lens and the imaging lens (MVPLAPO × 1, Olympus). The optical filter unit consisted of fluorescence filters with the center wavelengths of 600 nm, 632 nm, 650 nm and 680 nm (FF01-600/14-25, FF02-632/22-25, FF01-650/13-25, and FF01-680/22-25, Semrock).

### Image acquisition and processing with the device

After setting the prepared LNs in the device, the maximum value of fluorescence intensity was analyzed, while using image processing to eliminate the autofluorescence of backgrounds. It took a few minutes from tissue dissection to measurement completion.

### Image processing of autofluorescence removal with the device

To compare between two types of methods to eliminate the autofluorescence of background tissues, we acquired fluorescence images with the differential method [19] and the ratio method [20]. The basic principle of each image processing is described below.

#### Differential method

**Fig. 2:**
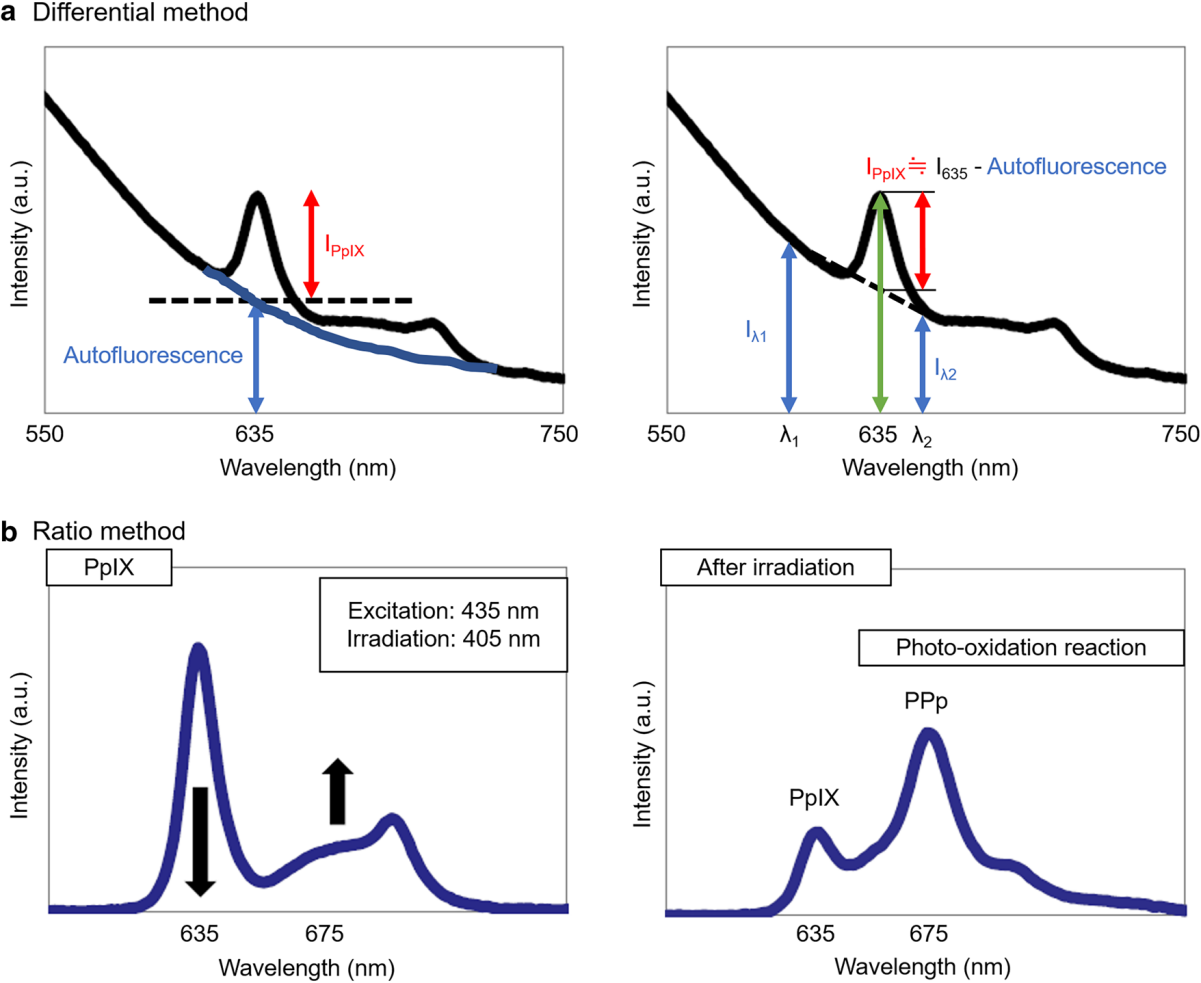
Basic principles of the differential method (**a**) and the ratio method (**b**). **a** The intensity of autofluorescent substances at 635 nm is estimated by a linear approximation of the spectrum. Protoporphyrin IX (PpIX) intensity at 635 nm is calculated by subtracting the autofluorescence intensity. **b** With continuous blue light irradiation, the photo-protoporphyrin (PPp) spectral peak around 675 nm increases and the PpIX spectral peak around 635 nm peak decreases. The increment in the intensity ratio of 675–635 nm before and after light irradiation indicates the presence of PpIX

In the differential method, PpIX fluorescence intensity against autofluorescence was quantitatively predicted using three wavelength regions (Fig. 2a). We focused on two sources of predominant autofluorescence of LN metastasis, derived from flavin adenine dinucleotide (FAD) and collagen. We employed optical filters with near the optimum center wavelength (CW) and full width at half maximum (FWHM) as follows: the CW of 600 nm with FWHM of 14 nm, 632 nm with FWHM of 22 nm, and 650 nm with FWHM of 13 nm. The wavelength of excitation light was 405 nm.

#### Ratio method

The ratio method utilizes photo-induced oxidization-assisted PpIX detection based on photoconversion effect of PpIX to photo-protoporphyrin (PPp) by blue light irradiation. There is a spectral peak of PPp at around 675 nm, while the peak at 635 nm in PPIX is decreased following blue light irradiation. The intensity ratio of 670 to 635 nm, thus, is increased in PpIX following light irradiation (Fig. 2b), while those in other fluorescent chromophores such as FAD and collagen remain unchanged. The wavelengths of excitation and irradiation light were 435 nm and 405 nm, respectively. The irradiated light fluence was set to 2 J/cm^2^. For ratio images, edges of the LNs were cut off to exclude them from the analysis target. There are two reasons for the process: first, the unexpected condensed light to the edges caused errors on the analysis of the fluorescence intensity. Second, since it requires a few tens of seconds for the photoconversion, the samples shrank during measurement, which was unfavorable for the analysis particularly in the edges.

As a sequence, first, differential imaging was performed under 405-nm excitation. Subsequently, for ratio imaging, an image was obtained under 435-nm excitation, followed by light irradiation at 405 nm, and an image after the photo-oxidation reaction was obtained at 435-nm excitation.

### Statistical analysis

JMP 14 (SAS Institute Inc., Cary, NC, USA) was used for all data analysis. Statistical significance was analyzed using Mann–Whitney *U* test. A value of *P* < 0.05 was considered as statistically significant.

## Results

**Fig. 3:**
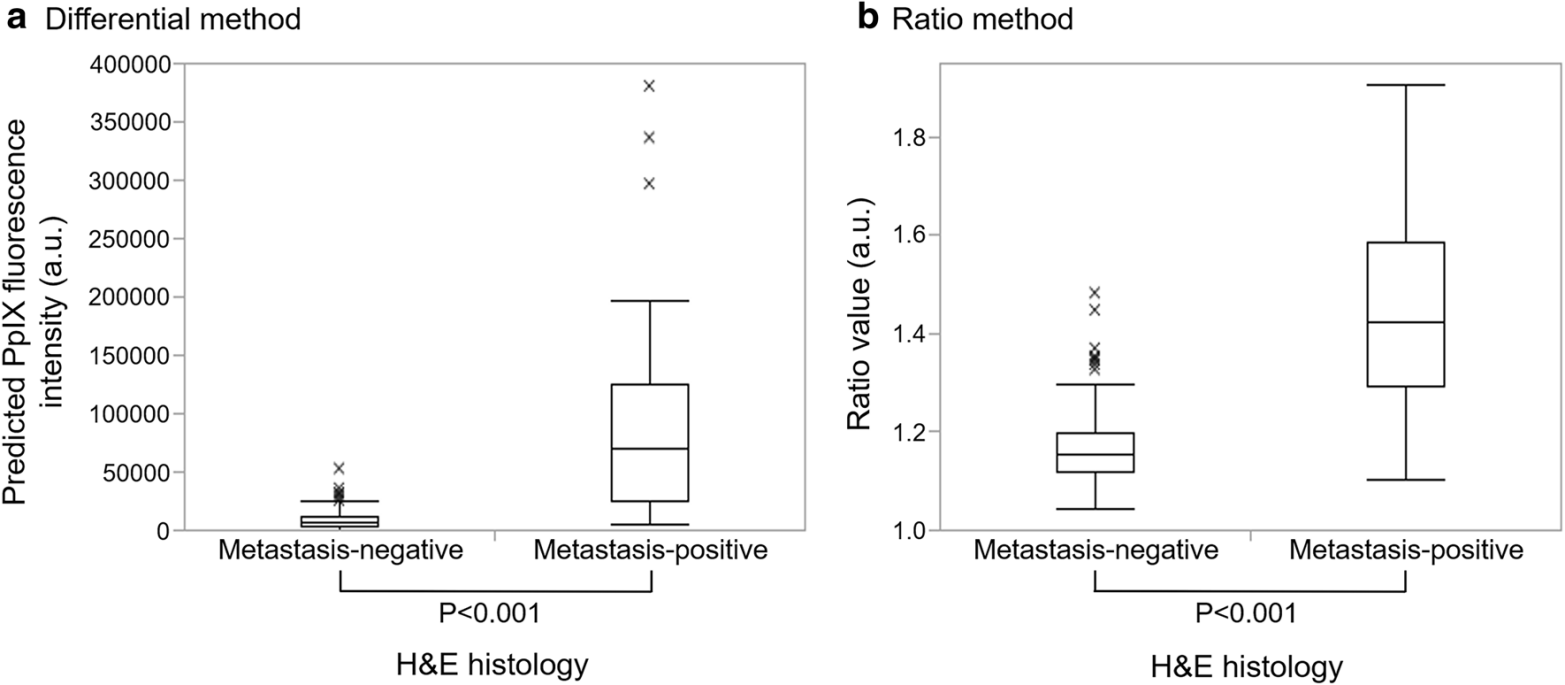
Comparison of intensity values between metastasis-positive and -negative LNs in the differential method (a) and ratio method (b). Metastatic LNs showed significantly higher values than non-metastatic LNs in each method (*P* < 0.001, respectively). *N* = 41 for metastasis positive and *N* = 282 for metastasis negative of the total 323 LNs from 64 patients

**Fig. 4:**
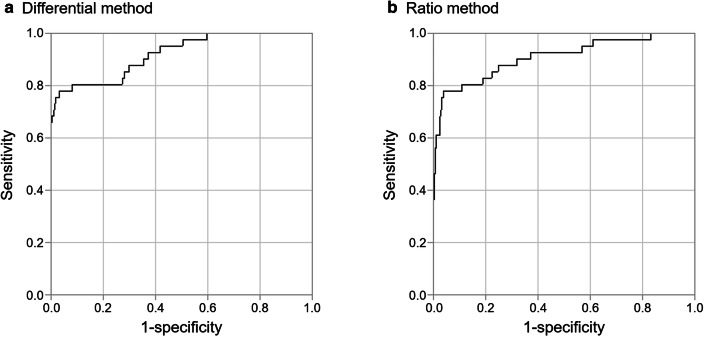
Receiver operating characteristic (ROC) curve analysis in the differential method (**a**) and ratio method (**b**). Areas under the curves (AUCs) were 0.921 (95% CI, 0.853–0.959) and 0.909 (95% CI, 0.830–0.953), respectively. *N* = 41 for metastasis positive and *N* = 282 for metastasis negative of the total 323 LNs from 64 patients

We first show the results of automated analysis of LN metastasis with the differential and ratio methods using the device for 323 LNs from 64 patients. In both methods, metastatic LNs showed significantly higher values than non-metastatic LNs (*P* < 0.001) (Fig. 3). The receiver operating characteristic (ROC) curve of each method is shown in Fig. 4. The areas under the curves (AUCs) of the differential method and ratio method were 0.921 and 0.909, respectively. Based on the calculated cutoff value as a threshold for each method, the sensitivity, specificity, and accuracy of classification with the differential method were 78.0%, 96.8%, and 94.4%, while those with the ratio method were 78.0%, 96.1%, and 93.8%, respectively **(**Supplementary Table 1).

Next, we compared the results for 211 LNs (included in the 323 LNs above) between the device and manual examination with fluorescence microscopy. In the device detection, the cutoff values calculated from the results for the above-mentioned 323 LNs were used as the thresholds. The fluorescence microscopy images were evaluated by three surgeons who were skilled at examining PpIX fluorescence in cancer tissues. When the opinions were different among them, they were decided on either finding as the result of discussion. Table 2 shows the results of analysis by device and stereoscopic examination. The sensitivity, specificity, and accuracy of classification with the differential method were 84.2%, 96.9%, and 95.7%, while those with the ratio method were 84.2%, 96.4%, and 95.3%, respectively. The sensitivity, specificity, and accuracy of the classification based on stereoscopic examination were 78.9%, 95.3%, and 93.8%, respectively. The diagnostic accuracy of each method with the device was comparable to that of manual examination in the conventional fluorescence microscopy.

**Table 2:** Comparison of metastasis detection results between the device and stereoscopic examination with fluorescence microscopy

	H&E histology		Total
	Metastasis positive	Metastasis negative	
Differential method			
Positive	16	6	22
Negative	3	186	189
Total	19	192	211
Ratio method			
Positive	16	7	23
Negative	3	185	188
Total	19	192	211
Stereoscopic detection			
Positive	15	9	24
Negative	4	183	187
Total	19	192	211

*n* = 211 LNs from 42 patients

**Fig. 5:**
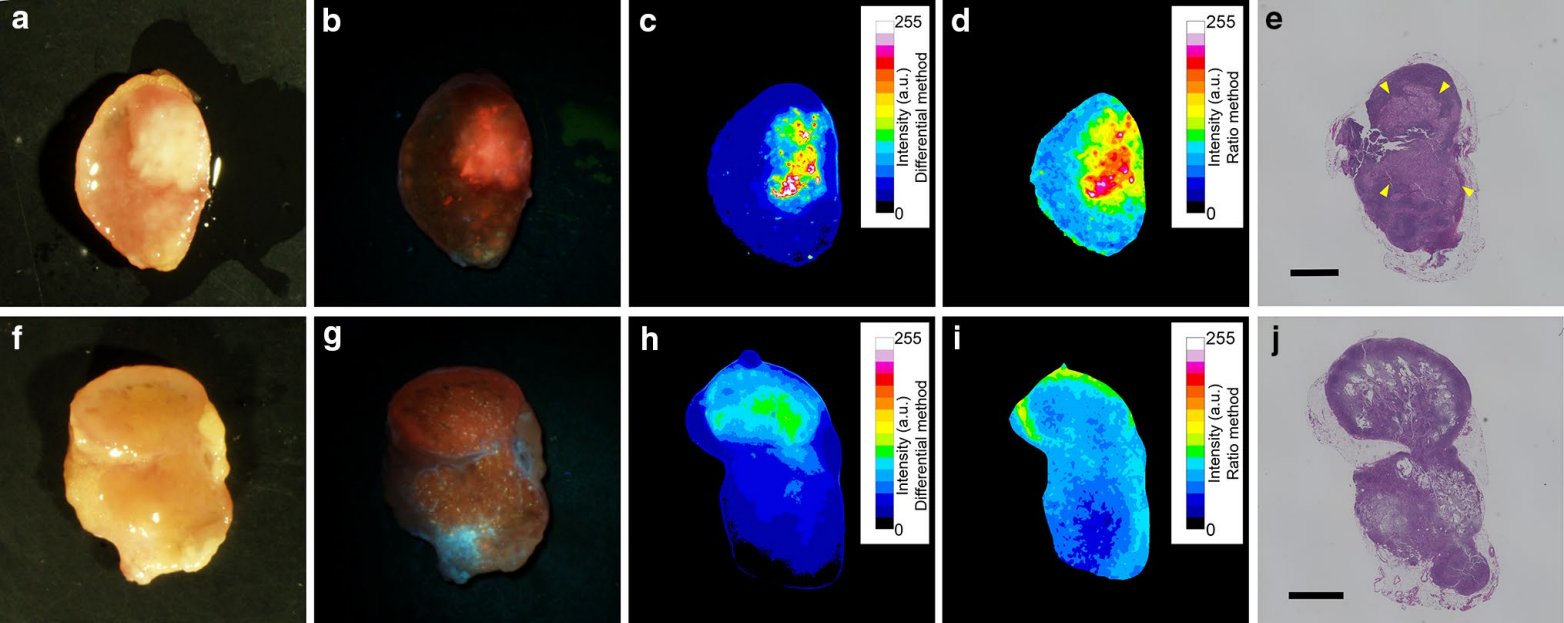
Representative images of metastatic (**a**–**e**) and non-metastatic (**f**–**j**). LNs (**a**) and (**f**) are white light images. **b** and **g** are fluorescence images acquired with conventional fluorescence microscopy. **c**, **h** and **d**, **i** are resultant images after applying differential method and ratio method processing, respectively. The intensity value was normalized in each method, and the fluorescence distributions are displayed with color mapping (**c**, **d**, **h**, **i**). H&E-stained section of each LN is shown (**e**, **j**). Arrowheads indicate metastatic lesion (**e**). Scale bar; 2 mm

Representative images of metastatic and non-metastatic LNs are shown in Fig. 5. In the metastatic LN image acquired with conventional fluorescence microscopy (Fig. 5b), distinct red fluorescence of PpIX accumulated in the metastatic lesion can be recognized. After applying the differential method and ratio method processing, a strong signal from PpIX is also observed for each (Fig. 5c, d). This LN was correctly diagnosed as metastasis positive with the two methods. In the non-metastatic LN image acquired with conventional fluorescence microscopy, red fluorescence with relatively strong intensity is shown (Fig. 5g); thus, it can be difficult to discriminate metastatic and non-metastatic with stereoscopic examination. After applying the two methods (Fig. 5h, i), the fluorescence intensity of PpIX was below the thresholds and the result for the device was metastasis negative. Referring to the H&E-stained slide (Fig. 5j), this fluorescence was considered to be derived from nonspecific accumulation of PpIX. Hence, this device enables quantitative evaluation of PpIX fluorescence intensity objectively and quickly.

## Discussion

We have reported that fluorescence diagnosis using 5-ALA has high diagnostic power to detect LN metastasis in gastrointestinal malignancies. However, the 5-ALA-induced fluorescence diagnosis has been based on visual intensity of PpIX fluorescence; thus, stereoscopic examination can include inter- and intra-observer variability due to cognitive difference. Therefore, we introduced automated detection using the device to address the issue. We have already developed methods to eliminate background fluorescence and we combined them for automated detection with the device.

From the analysis of 323 LNs, metastatic LNs showed significantly higher intensity values in both methods to eliminate autofluorescence. In addition, high accuracies were achieved through device analysis based on the values. From these results, we could demonstrate the validity of the detection based on the quantitative evaluation of PpIX fluorescence intensity. Furthermore, in the analysis of 211 LNs, this device showed diagnostic performance comparable to that of stereoscopic detection, while maintaining rapidity and objectivity.

We analyzed error images retrospectively. Even considering the small number of metastasis-positive LNs, there is still room for improvement of the accuracy. When the metastatic lesions having less than 2 mm in diameter were defined as micro-metastasis, their fluorescence intensities tended to be weaker than macro-metastasis lesions. In both the differential and ratio methods, more than half of the false-negative instances were micro-metastasis lesions in analyzing 323 LNs. In fact, of the 6 micro-metastasis lesions, 5 were false negative by at least one of the methods.

In comparison, there were 9 and 11 false-positive instances of 41 metastatic LNs, respectively, with the differential and ratio methods in analyzing 323 LNs. In the false-positive instances, it is possible that the fluorescence derived from the site of inflammation where PpIX is accumulated nonspecifically was falsely detected. Our results indicate that it is difficult to completely discriminate non-specific accumulation in inflammation only by quantitative evaluation of fluorescence intensity. Some researchers reported that PpIX accumulation in inflamed sites was related to intracellular iron metabolism [21, 22], but the detailed mechanism is still unclear. We require further investigation on the mechanism to reduce the incidence of false positive due to this issue.

For the non-specific accumulation of PpIX, the fluorescence in lymphoid follicles has been manually eliminated by the observers based on the morphological features [9]. Since this study is based on quantitative evaluation of PpIX and removal of autofluorescence, no interventional processing for removal of the follicle fluorescence was performed. As a more advanced model, we need to consider image processing to automatically remove the follicle fluorescence. In connection with automatic image analysis, deep neural network (DNN), one of the machine learning approaches, has demonstrated outstanding performance in automated image-recognition applications [23, 24]. Therefore, it may be effective to remove follicle fluorescence morphologically using the DNN analysis.

In this study, we applied two types of methods to eliminate autofluorescence. Our results showed that the diagnostic power with the differential method was slightly better than that of the ratio method. Comparing the light irradiation time between the two methods, the ratio method requires about a few tens of seconds to minutes for photoconversion. In comparison, in the differential method, a small number of image acquisitions allow faster imaging, short total exposures that are safe for the specimen, enabling in vivo imaging. However, in vivo analysis of deep LNs in adipose tissue is difficult considering the penetration depth of the excitation wavelength. Since this study is an ex vivo analysis, adverse effects on human bodies due to relatively long irradiation in the ratio method can be ignored. Although it may be better to use them depending on the situation, it is difficult to determine which method is better for detecting LN metastasis based on this study alone. Since the instances which were misdiagnosed in each method were almost the same in this study, significant improvement in accuracy cannot be expected with the combined method. Overall, this study showed that this device has a potential to automatically detect PpIX fluorescence while removing autofluorescence.

A potential limitation is the small sample size. Multicenter clinical research is desirable to accumulate more data. We consider that clinical trials can be further accelerated by introducing automatic diagnosis using the device. Another limitation is detection of micro-metastasis. As mentioned above, since micro-metastatic lesions show the weak intensity of PpIX fluorescence, lowering the thresholds to detect them inevitably increases the incidence of false positive. It would be ideally desirable to reduce both false-positive and false-negative incidence accounting for the application to intraoperative diagnosis, but it is difficult to achieve with this method diagnosing according to the thresholds of fluorescence intensity. In clinical practice related to gastric cancer, there is no defined regulation in considering a small metastatic lesion in SN as a prognostic factor. In breast cancer, it has been reported that the long-term prognosis is not significantly affected by omitting additional axillary dissection for SN metastasis smaller than 2 mm [25]. Even in gastric cancer, if the relationship between the size of the metastatic lesion and long-term prognosis is more clarified, it may be effective to set a threshold according to the size.

We consider the potential importance of this system in clinical practice as follows. First, this device enables detection of LN metastasis without subjectivity. Second, although it is necessary to further increase the number of samples, it has the advantage of improving measurement accuracy and accelerating clinical research by constructing a simple automatic measurement system. Third, an automatic diagnostic system has the potential to introduce intraoperative diagnosis even in facilities without pathologists in the future. In addition, we believe that this approach can be applied to analyze PpIX fluorescence images obtained from various types of 5-ALA-introduced samples. To bring the methods closer to clinical practice, we require further investigation.

## Conclusions

Our results show that this device automatically detects LN metastasis with high accuracy, which was comparable to that of stereoscopic examination. Overall, this study demonstrates that our developed device for automated detection of metastatic LNs using 5-ALA can be a useful tool for intraoperative diagnosis.

## Electronic supplementary material

Below is the link to the electronic supplementary material.

Supplementary material 1 (PDF 174 kb)
