# Supplementary material for: 5-ALA-assistant automated detection of lymph node metastasis in gastric cancer patients
Source: Gastric Cancer. 2020 Feb 11;23(4):725–33. doi: 10.1007/s10120-020-01044-w (PMC7305096; doi:10.1007/s10120-020-01044-w)
Supplement: Supplementary file 1 — Supplementary material 1 (PDF 174 kb) [file 10120_2020_1044_MOESM1_ESM.pdf]

Supporting Information

5-ALA-assistant automated detection of lymph node metastasis in gastric cancer patients

Tatsuya Matsumoto[1,2], Yasutoshi Murayama[1], Hisataka Matsuo[1,2], Kengo Okochi[3], Naotaka Koshiishi[3], Yoshinori Harada[2], Hideo Tanaka[2], Tetsuro Takamatsu[2,4], Eigo Otsuji[1]

[1]Division of Digestive Surgery, Department of Surgery, Kyoto Prefectural University of Medicine, 465 Kajiicho, Kawaramachi-Hirokoji, Kamigyo-ku, Kyoto 6028566, Japan

[2]Department of Pathology and Cell Regulation, Kyoto Prefectural University of Medicine, 465 Kajiicho, Kawaramachi-Hirokoji, Kamigyo-ku, Kyoto 6028566, Japan

[3]Ushio Inc., 6409 Moto-Ishikawa-cho, Aoba-ku, Yokohama, Kanagawa 2250004, Japan

[4]Department of Medical Photonics, Kyoto Prefectural University of Medicine, 465 Kajiicho, Kawaramachi-Hirokoji, Kamigyo-ku, Kyoto 6028566, Japan

Supplementary table 1 Results of automated metastasis-detection with the differential and ratio methods

		H&E histology		
		Metastasis-positive	Metastasis-negative	Total
Differential method	Positive	32	9	41
	Negative	9	273	282
	Total	41	282	323

		H&E histology		
		Metastasis-positive	Metastasis-negative	Total
Ratio method	Positive	32	11	43
	Negative	9	271	280
	Total	41	282	323

n = 323 LNs from 64 patients
